# A scoping review of classification schemes of interventions to promote and integrate evidence into practice in healthcare

**DOI:** 10.1186/s13012-015-0220-6

**Published:** 2015-03-03

**Authors:** Cynthia Lokker, K Ann McKibbon, Heather Colquhoun, Susanne Hempel

**Affiliations:** Department of Clinical Epidemiology and Biostatistics, Health Information Research Unit, McMaster University, CRL Building, 1280 Main Street West, Hamilton, L8S 4 K1 ON Canada; Department of Occupational Science and Occupational Therapy, University of Toronto, 160-500 University Avenue, Toronto, ON M5G 1 V7 Canada; RAND Corporation, 1776 Main Street, m4339, Santa Monica, 90407 CA USA

**Keywords:** Knowledge translation, Implementation science, Classification, Dissemination and implementation, Scoping review

## Abstract

**Background:**

Many models and frameworks are currently used to classify or describe knowledge translation interventions to promote and integrate evidence into practice in healthcare.

**Methods:**

We performed a scoping review of intervention classifications in public health, clinical medicine, nursing, policy, behaviour science, improvement science and psychology research published to May 2013 by searching MEDLINE, PsycINFO, CINAHL and the grey literature. We used five stages to map the literature: identifying the research question; identifying relevant literature; study selection; charting the data; collating, summarizing, and reporting results.

**Results:**

We identified 51 diverse classification schemes, including 23 taxonomies, 15 frameworks, 8 intervention lists, 3 models and 2 other formats. Most documents were public health based, 55% included a literature or document review, and 33% were theory based.

**Conclusions:**

This scoping review provides an overview of schemes used to classify interventions which can be used for evaluation, comparison and validation of existing and emerging models. The collated taxonomies can guide authors in describing interventions; adequate descriptions of interventions will advance the science of knowledge translation in healthcare.

**Electronic supplementary material:**

The online version of this article (doi:10.1186/s13012-015-0220-6) contains supplementary material, which is available to authorized users.

## Background

The advancement of the science of knowledge translation or how to most effectively promote and support the use of evidence in health and healthcare policy and practice is challenged by the plethora of terms, models and frameworks and heterogeneous interventions employed in the field. Broadly, knowledge translation is the synthesis, dissemination, exchange and ethically sound application of knowledge to improve health [1]. In this field and the overlapping fields of quality improvement, research utilization, behaviour change, dissemination and implementation (to name but a few), descriptions of interventions and their content lack consistency and mutual understanding among stakeholders [2]. Potentially, key details of interventions are often not reported [3-8], partially due to limitations on space in articles [8], the complexity of some of the interventions [9] and a lack of agreement what these key details are. Without sufficient description, generalizability across and replication of interventions are difficult. Combining evidence from a number of studies in systematic reviews becomes impractical when interventions are not described adequately and increases the challenge to determine which elements are important for success.

To address these challenges of reporting, many authors have provided ways to classify or describe interventions. One approach is the development and dissemination of reporting guidance of details to include in publications. Prominent examples are the Consolidated Standards of Reporting Trials (CONSORT) [10] and the Template for Intervention Description and Replication (TIDieR) [11] which focuses on describing interventions with enough detail to allow for replication. However, reporting guidelines come from a variety of disciplines in health and social science and are based on interventions most commonly encountered in their individual field of research. These interventions may differ widely and are oriented towards different classification schemes or intervention ‘taxonomies’.

A classification scheme allows for identification and description of various entities and grouping by similarity and a number of approaches other than reporting guidance have been taken in the development of such schemes. Strictly, a taxonomy is a hierarchical array of controlled vocabulary, with broader and narrower related terms, whose main function is to remove ambiguity of a concept [12] (e.g., Systematized Nomenclature of Medicine [SNOMED], a comprehensive, multilingual, clinical terminology). Ideally, taxonomies need to be adaptive, especially with changing language. Stavri and Michie provide a good description of six types of classification systems (nomenclatures, ordered sets, hierarchical, matrices, faceted and social categorizations) and make a case for a hierarchical taxonomy of behaviour change techniques [13]. Damschroder et al. published a Consolidated Framework for Implementation Research (CFIR), comprising a menu of constructs associated with effective implementation of interventions, in an attempt to structure and consolidate competing approaches [14]. The CFIR has also been adapted to other topic areas [15]. To date, a list of intervention types summarizing studies registered in the Cochrane Effective Practice and Organization of Care (EPOC) Group is widely used [16], although it was not designed to function as a taxonomy. Each of these examples represents different ways in which interventions can be classified. Given that few meet the traditional definition of taxonomy, we refer to them in this paper as classification schemes and include approaches such as lists, taxonomies, frameworks and checklists.

In order to encourage the use of classification schemes, authors and designers of interventions need to better understand the range of classification schemes available. We performed a scoping review of the literature and contacted content experts for classification schemes used to describe interventions that promote and integrate evidence into health practices, systems and policies across a number of fields including public health, clinical medicine, nursing, policy, behaviour science, improvement science and psychology. Broadly, classification schemes could include frameworks, taxonomies, terminologies, glossaries, nomenclatures or reporting guidance. We use ‘classification scheme’ to describe any of these modes of classifying or describing interventions. The objective of the review was to gather and summarize classification schemes according to their content, the context for which they were developed, the method of development, whether original or based on an existing scheme, and if or how peer review and theory specifically were used for development. This will allow authors to access a summary of existing schemes in one place, for evaluation and comparison of schemes, and to guide validation of existing models.

## Methods

Scoping reviews aim to map the literature on a topic [17]. We have utilized the five-stage methodological process as outlined in Arksey and O’Malley [17] and the enhancements proposed by Levac et al. [18] to conduct our scoping review. The five stages undertaken were identifying the research question; identifying relevant literature; study selection; charting the data; and collating, summarizing, and reporting results. We chose a scoping review methodology and limited our search parameters as we knew that a really broad search would not be feasible given the lack of index terms and consistency in naming and describing classification schemes, due to the broad number of approaches to developing such schemes.

### Identifying the research question

In 2012, a group of researchers participated in an international consensus meeting on knowledge translation terminology and developed a draft simplified model of interventions [19]. One of the questions that evolved over the course of the meeting was the following: what classification schemes (frameworks, terminologies, taxonomies, glossaries, nomenclatures, etc.) are available to guide design or description of interventions? Other related questions also became evident. How do these schemes describe interventions? How were they derived? How do they compare? The scoping review working group (CL, HC, KAM, SH) further refined the question to include domain areas of evidence-based practice, dissemination and implementation, quality improvement, behaviour change and other related knowledge areas.

### Identifying and selecting articles

When developing our inclusion and exclusion criteria, we took an iterative approach as described by Levac et al. [18], starting with frameworks and classification schemes submitted by participants of the international consensus meeting on knowledge translation terminology. Participants were asked to provide frameworks related to promoting and integrating evidence into practice that they had developed or used in their work. These frameworks came from researchers in the fields of knowledge translation, behaviour change, quality improvement and policy. We determined which of the frameworks provided a scheme, framework or model that could be used to classify or describe interventions and used them to develop and refine our searching plan as well as our inclusion and exclusion criteria. One submission, a review of behaviour change interventions by Michie et al., identified 19 existing schemes which were used to develop their new framework for interventions aimed at behaviour change [20]. The consensus of the searching experts on our team (AM and SH) was that the Michie [20] review covered important behaviour change classification schemes up to 2009 and could form the starting point for our search strategy. We updated the search from the Michie review to 2013. Since the search focused on behaviour change, we added terms related to knowledge translation to capture more broadly classification schemes across content areas including dissemination and implementation, knowledge translation, quality improvement, knowledge transfer and research utilization.

We searched MEDLINE, PsycINFO and CINAHL with the terms: classification [index term] OR taxonomy.mp combined with: (behavior* change or behavior* intervention or behavior* change intervention) from 2009 to May 2013 to update the search from Michie [20]; and from inception to May 2013, we combined classification [index term] OR taxonomy.mp combined with knowledge translation terms intended to capture the field (implementation OR knowledge translation OR quality improvement OR knowledge transfer OR research utilization OR dissemination). These terms may not be exhaustive, but a number of them are frequently used in intervention literature [21]. We did not add the terms ‘framework’ or ‘model’ since these are used widely in the literature without clear definitions and they would reduce the specificity of our searches (increasing number of false positives).

Grey literature searches included the following websites related to promoting and integrating evidence into health practices, systems and policies: KT Canada Clearinghouse (http://ktclearinghouse.ca/tools/uncategorized); Implementation Central (http://www.implementationcentral.com/index.html); Alberta Innovates Health Solutions (http://www.aihealthsolutions.ca/outreach-learning/ktn/resources/); Research and Development Resource Database, University of Toronto; CFIR Wiki http://cfirwiki.net/wiki/index.php?title=Main_Page; NCCMT (May 23, 2013) Registry http://www.nccmt.ca/registry/index-eng.html; and Netting the Evidence.

We reviewed bibliographies of, and citations to, identified documents for other potential citations. We relied on substantial bibliography checking to maximize retrievals because of lack of indexing terms and multiple definitions and usage of terms. Titles and abstracts were screened independently in duplicate by AM and CL. For studies that passed initial screening, full-text articles were assessed independently by AM and CL to determine inclusion. Any disagreements were adjudicated by HC.

### Inclusion criteria

Articles, books or reports with frameworks (a basic underlying structure of a system or body of knowledge), glossaries (list of terms with definitions or descriptions), taxonomies (hierarchical array of controlled vocabulary, with broader and narrower related terms), terminologies (list of terms associated with a specific body of knowledge grouped in a logical order), or nomenclatures that, at a minimum, provided categories or ways to classify or describe interventions aimed at integrating evidence into practice and policy were included. There had to be a deliberate attempt to classify or describe interventions or efforts/activities such as lists of types of interventions (e.g. EPOC [16]) or components or ‘domains’ of interventions (e.g. behaviour change wheel [20]). Policy articles, which typically do not use the language of ‘intervention’, were included if the focus was on offering ways of framing interventions or actions. We excluded non-English language documents; those that mentioned ‘intervention’ in a framework without further elaboration on any descriptions of the interventions or intervention components; descriptions of classification schemes that were reported in earlier publications; and tools for designing interventions without descriptions of components.

### Charting the data

AM, CL and HC iteratively designed the data collection forms as data were being abstracted for the first number of articles [18]. Modification of the data forms continued until we reached saturation on content. Ultimately, the following descriptive details were abstracted from the articles by CL: ‘focus’ (what are the authors describing/classifying), context (discipline or field of study), objective of the article; a description of the classification scheme (e.g., list, framework, taxonomy, other); composition of the scheme (in terms of types of elements included); a free-text description of the elements of the classification scheme (e.g., levels, constructs); method of development (e.g., review, consensus); theoretical basis; use of knowledge users in development; and whether the scheme was piloted or tested, new or based on existing scheme and peer reviewed. These data were entered into charts in a database for collation, summarization, presentation and reporting.

## Results

### Article selection

Titles and abstracts of over 1,700 items from group members, searches and reference lists were screened; 134 full-text items were assessed, and 51 articles that provided classification schemes were identified and summarized. Four articles could not be located (Additional file 1). A summary of the objectives of the publications contacting the classification schemes is available in Additional file 2.

### Collating, summarizing and reporting the results

A recent increase in classification schemes was found, with more than half of them published since 2010. Most classification schemes came from the UK and USA; 23 primary authors were from the USA, 17 from the UK, 7 from Canada and 2 each from Australia and the Netherlands. Seventeen schemes were intended for interventions in general while 34 focused on a specific context. The majority of the 51 schemes were in public health, namely health promotion and behaviour change in this domain. Patient safety, policy, nursing and social work were also represented.

Methods of development included coding of documents, literature and document reviews, and use of expert panels. Twenty-eight of 51 reports included some form of a document review; 14 did not report how the classification scheme was derived. Forty-two articles were peer reviewed; the remainder were contained in reports for governments or agencies (e.g., [22-26]) or as guides to interventions for authors [16] or searchers [27] or book chapters [28,29]. A number of authors enlisted knowledge users in the development of the classification schemes [23,30-39], while others employed strategies to pilot test the schemes [20,23,30,32-38,40-47]. One such pilot test is currently underway [48].

Some authors derived new classification schemes, while 25 extended existing schemes. Of existing schemes, three publications adapted or extended the EPOC list of interventions. These included Health Systems Evidence [27], Shojania et al. [49] and Mazza et al. [44]. Three publications adapted or extended behaviour change techniques [40] to a number of specific areas including smoking cessation [42], physical activity and healthy eating [41] and alcohol consumption [45]. Another approach was to map or combine a number of models or frameworks and consolidate them [14,20,32,38,39,49,50]. Notable examples are the behaviour change wheel [20] and the CFIR [14]. Eighteen of the schemes were theory based; of these, 13 were based on existing schemes. Of the 21 new schemes, 4 were theory based. In 5 cases, we were unable to determine if the scheme was new or based on previous models.

The classification schemes fall into a number of types based on the descriptors given by the authors. In the absence of a clear approach to subdividing 51 frameworks and to ensure that we avoided one unwieldy table, we attempted to divide the classification schemes into the following categories, largely based on what terms authors used to describe their scheme: those that are essentially lists, taxonomies, frameworks and other. Eight classification schemes were lists of interventions or behaviour change techniques, a checklist or a catalogue (Table 1); 23 were described as taxonomies by the authors (Table 2); 15 were described as frameworks (Table 3); 1 as a ladder [25], 3 as models [37,38,51] and 1 as a reporting guideline [52] (Table 4). Although ‘taxonomy’ generally refers to a hierarchical system of classification, use of the term by authors was not limited to this structure. However, we categorized them in the taxonomy class since that is how the authors described them.

**Table 1:** **Lists to classify interventions (** ***n*** **= 8)**

**Classification scheme**	**Context**	**Focus of scheme**	**Methodology**	**Peer reviewed**	**Knowledge users**	**Piloted or tested**	**Theory based**
Abraham 2011 [28]	Health promotion	List of behaviour change techniques	Not reported	No	No	No	Yes
AHRQ [26]	Patient safety	List of approaches to improve safety	Not reported	Not reported	Not reported	Not reported	No
Albrecht 2013 [48]	General	Checklist for assessing quality of KT interventions	Literature review	Yes	No	Underway	No
Bartholomew 2011 [29]	Health promotion	Methods for changing behaviours	Not reported	No	No	Not reported	Yes
CPHI 2001 [24]	Public health	Includes a catalogue of strategies by who, when, how of getting research into policy	Survey	No	No	No	No
EPOC 2010 [16]	Health	Data collection checklist that includes a list of interventions for inclusion in Cochrane reviews	Not reported	No	Not reported	Not reported	No
Health Systems Evidence 2013 [27]	Policy, health systems	List of health system arrangements and implementation strategies in Health Systems Evidence web resource	Not reported	No	No	No	No
Powell 2012 [39]	Health	Compilation of implementation strategies	Literature review	Yes	Yes	No	No

**Table 2:** **Taxonomies to classify interventions (** ***n*** **= 23)**

**Classification scheme**	**Context**	**Focus of scheme**	**Methodology**	**Peer reviewed**	**Knowledge users**	**Piloted or tested**	**Theory based**
Abraham 2008 [40]	General	Theory-linked taxonomy of behaviour change techniques	Review, mapping,	Yes	No	Yes	Yes
Carlson 2010 [53]	Health, policy	A taxonomy of performance-based, health outcomes—reimbursement schemes	Literature review	Yes	No	No	No
Dogherty 2010 [54]	Health, nursing	Taxonomy of facilitation interventions/strategies and facilitator role	Literature review	Yes	No	No	No
Embry 2008 [55]	Psychology	The 52 evidence-based kernels that produce reliable effects on behaviour	Not reported	Yes	No	No	No
Evenboer 2012 [36]	Social work, youth	Taxonomy of care for youth	Interviews, chart reviews, expert panel,	Yes	Yes	Yes	No
Galbraith 2011 [43]	Health, HIV/AIDS prevention	Taxonomy of core elements of evidence-based behavioural interventions	Literature review	Yes	No	Yes	No
Geller 1990 [56]	Public health, injury control	Taxonomy of behaviour change strategies to guide intervention development and evaluation in injury control	Not reported	Yes	No	No	Yes
Gifford 2013 [57]	Health, nursing	Taxonomy of leadership and management behaviours	Qualitative interviews	Yes	No	No	Yes
Hardeman 2000 [58]	Public health, obesity	A taxonomy of behaviour change programs for classifying models, change methods, and modes of delivery for prevention of weight gain	Literature review	Yes	No	No	No
Lamb 2011 [35]	Health; public health	Taxonomy to classify and describe fall-prevention interventions	Literature review, consensus	Yes	Yes	Yes	No
Leeman 2007 [50]	Nursing	Taxonomy of methods for implementing change in practice	Literature review and content analysis	Yes	No	No	Yes
Lowe 2011 [59]	Health; medication use	Taxonomy of interventions for improving consumer medication use	Literature review and thematic analysis	Yes	No	No	No
Mazza 2013 [44]	Guideline implementation	Taxonomy of implementation strategies	Amendment of EPOC	Yes	No	Yes	No
Michie 2012 [45]	Public health	Taxonomy of behaviour change techniques for reducing excessive alcohol consumption	Coding of documents	Yes	No	Yes	No
Michie 2008 [46]	Behaviour change	Initial list of behaviour change techniques	Literature review, brainstorming	Yes	No	Yes	Yes
Michie 2011 [41]	Public health	Behaviour change techniques for physical activity and healthy eating behaviours	Iterative refining; document coding	Yes	No	Yes	Yes
Michie 2011 [42]	Public health	Behaviour change techniques used in individual behavioural support for smoking cessation	Coding of two key documents	Yes	No	Yes	Yes
Reisman 2005 [60]	General	A cross-disciplinary taxonomy of transfer of technologies	Literature review	Yes	No	No	No
Schulz 2010 [34]	General	Taxonomy of delivery characteristics and content and goals of interventions	Literature review	Yes	Yes	Yes	No
Shojania 2004 [49]	Quality improvement	Taxonomy of quality improvement strategies	Literature review	Yes	No	No	No
Taylor 2011 [31]	Patient safety	Taxonomy of contextual features important to the effectiveness of patient safety practice interventions	Expert panel, literature review	Yes	Yes	No	No
Walter 2003 [61]	General	Cross-sectorial taxonomy of interventions to increase the impact of research	Literature review	Yes	No	No	Yes
West 2006 [62]	Public health	Includes a simple taxonomy of approaches designed to influence behaviour patterns	NR	Yes	NR	No	NR

**Table 3:** **Frameworks (** ***n*** **= 15) to classify interventions**

**Classification scheme**	**Context**	**Focus of scheme**	**Methodology**	**Peer reviewed**	**Knowledge users**	**Piloted or tested**	**Theory based**
Best 2008 [30]	Oncology	Knowledge integration framework for cancer control	Expert panels, literature review and concept mapping	Yes	Yes	Yes	No
Cane 2012 [33]	General	Refinement of the theoretical domains framework	Card sorting and cluster analysis	Yes	Yes	Yes	Yes
Century 2012 [63]	Education and social sciences	Two frameworks for describing implementation and for factors that affect implementation	Literature review	Yes	No	No	No
Cohen 2000 [64]	Public health, HIV prevention	Taxonomy of HIV interventions across individual and structural levels	Not reported	Yes	No	No	No
Czaja 2003 [47]	Behavioural interventions (Alzheimer’s Disease)	Describing and decomposing complex psychosocial and behavioural interventions	Mapping interventions onto framework	Yes	No	Yes	Yes
Damshroder 2009 [14]	General	Consolidated Framework For Implementation Research (CFIR)	Literature review; snowballing, synthesis of theories and frameworks	Yes	No	No	Yes
Dixon 2010 [23]	Behaviour change	Competences to deliver interventions to change lifestyle behaviours that affect health	Literature review, feedback	No	Yes	Yes	Yes
Dolan 2010 [22]	Policy	Checklist for policy-makers aimed at changing or shaping behaviour	Not reported	Not reported	Not reported	Not reported	Yes
Dy 2011 [32]	Patient safety	Patient safety practices	Literature review, consensus with experts	Yes	Yes	Yes	No
Goel 1996 [65]	Social science, retail pharmacies	Conceptual framework of factors that affect retail pharmacy prescribing and strategies for behaviour change	Literature review	Yes	No	No	No
Hendriks 2013 [66]	Public health	Behaviour change ball framework for public health and childhood obesity	Literature review	Yes	No	No	Yes
Lavis 2006 [67]	Policy, international	Framework for assessing country-level efforts to link research to action	Not reported	Yes	No	No	No
Michie 2011 [20]	Behaviour change	Behaviour change wheel comprising: a ‘behaviour system’ at the hub, encircled by intervention functions and then by policy categories	Literature review; development of new framework	Yes	No	Yes	Yes
Purdue 2005 [68]	Policy	Legal strategies for preventing cardiovascular disease	Not reported	Yes	No	No	No
Stirman 2013 [69]	General	Coding system for modifications and adaptions of interventions	Literature review	Yes	No	No	No

**Table 4:** **Other classification schemes to classify interventions (** ***n*** **= 23)**

**Classification scheme**	**Context**	**Focus of scheme**	**Methodology**	**Peer reviewed**	**Knowledge users**	**Piloted or tested**	**Theory based**
Greenhalgh 2004 [51]	General	Diffusion of innovations in health service delivery and organization	Literature review	Yes	No	No	No
Keller 2004 [37]	Public health	Intervention wheel for public health	Literature review, expert panel	Yes	Yes	Yes	No
Nuffeld 2007 [25]	Public health	Intervention ‘ladder’ of public health interventions from an ethical viewpoint	Not reported	No	No	No	No
Proctor 2013 [52]	Health	Recommendations for specifying and reporting implementation strategies	Not reported	Yes	No	No	No
Ward 2010 [38]	General	Knowledge transfer model with five elements: classify problem; context; knowledge; intervention; use	Literature review, fieldwork, revising	Yes	Yes	Yes	No

## Discussion

This scoping review of classification schemes used to describe interventions that promote and integrate evidence into health practices, systems and policies identified 51 diverse classification schemes across the areas of public health, clinical medicine, nursing, policy, social work, behaviour science and improvement science.

The tables which divide the classification schemes into lists, taxonomies and frameworks plus others represent different approaches that have been taken to classify interventions. We appreciate that categorizing the classifications schemes could be achieved in numerous ways; we have offered one approach. The schemes could also be categorized as those describing interventions to improve implementation of evidence-informed recommendations and those that improve the use of research evidence in policy decisions at the levels of clinical, health system or public health. There remains work to do in this field. There are many different types of classification schemes, and we do not yet have a clear sense of all potential groupings. Input from other experts in the various fields may provide further insights into how we might refine categories of schemes and how to classify them.

Ideally, classification schemes need to be responsive to developments in the field. Some authors have compiled lists of available types of interventions and derived categories based on similarities. The behaviour change wheel [20] approached interventions by developing 14 domains of theoretical constructs related to behaviour (e.g. knowledge, skills, social/professional role and identity, beliefs about capabilities). The number of schemes developed from previous models (*n* = 25) was similar to the number of new schemes (*n* = 21) for publications where we could discern this variable. This observation indicates that in some specific areas, there may be emerging consensus on ways to classify interventions (e.g., behaviour change techniques) but the number of new schemes indicates that, as a field, there is no broad agreement.

A number of the classification schemes were derived by reviewing the literature, compiling constructs or domains that are important to the description of the interventions under consideration and then testing the schemes to be sure that they were able to classify new interventions (e.g., [20,30,41,44]). Including knowledge users in the process allowed for stakeholder engagement and agreement in the process and outcomes. Of the 51 included schemes, 27% did not report on the method of development. While methods for the development of classification schemes are varied, with some including literature review, knowledge users, mapping of a number of schemes, use of theory and pilot testing, there is no best practice guide for scheme development. We also do not understand if the way in which a classification scheme is developed could have an impact on how it is used and by whom. Comparative analysis of different schemes, along with better understanding of the contexts in which each have been developed and used, could facilitate their development and use rates. Such analysis could include a multi-pronged approach including mapping of schemes to each other, content analysis and natural language processing techniques.

We also note that despite the large number of schemes being available, there is still an apparent problem of interventions not being reported well enough in the literature for effective application in another setting [3,6-8,52]. The value of having multiple schemes, a number of which are reporting guidance, does not seem to be moving the field towards a better understanding of interventions and the ability to compare outcomes of these interventions. More targeted efforts are needed, including more awareness and application of the different intervention classification schemes and the adoption and enforcing of appropriate reporting guidance by journals publishing intervention evaluations.

This scoping review aims to provide the basis for informing the field. The inventory can be used as a resource for researchers tackling the issue of terminology in the field, for evaluation and comparison of schemes, and to guide validation of existing and emerging models. Other authors are also attempting to synthesize models for the field with the goal of allowing researchers to identify and select the appropriate model for their work [70]. Our future work includes comparing the classification schemes to the recent simplified model of knowledge translation interventions [19] as one step in validating the applicability of the model. The inventory of classification schemes may also help researchers find a suitable one for their needs and avoid duplication or development of new schemes. Future studies could include comparative analysis of the 51 classification schemes to determine common and divergent terminologies and elements. It is difficult to discern the degree to which present classification schemes have been adopted by researchers and practitioners. Future research determining who uses the schemes and how and why they are used would be valuable especially for existing reporting guidance documents.

Other domains of knowledge have similar challenges with their classification schemes, some of which develop schemes based on need and task requirements. For example, the informatics domain has developed schemes that are used to report regional and national health statistics for comparison across disciplines (International Classification of Diseases, versions 9 and 10), billing purposes (SNOMED initially developed for laboratory procedures and the Current Procedures Terminology [CPT] for other health-related procedures) and indexing (U.S. National Library of Medicine, Medical Subject Headings). See http://www.ncvhs.hhs.gov/080221p4.pdf.

As with the informatics domain, we need to acknowledge that different models and classification schemes exist and that they were developed with different goals and starting points. To ensure that researchers and practitioners gain the maximum benefit from classification schemes, we need to make the goals and foundations of each scheme transparent and readily available and acknowledge that other classification schemes exist that could also inform use.

Our scoping review has a number of limitations. We include only English-language publications, and we focused our searches in the medical-centred literature and did not search general social science databases though the nursing and psychological fields are represented. Classification schemes are tools; and often, tools are not fully described in the scientific literature, making them difficult to detect through searches. We chose a scoping review methodology since we believed that a systematic search and review of classification schemes would be too challenging for our objectives. We wanted to gain an understanding of what kinds of classification schemes were available in the field rather than present a full complement of them. Our searches could have missed spelling variants, and our grey literature search was Canadian focused. We balanced these challenges with substantial bibliography checking, an alternate approach to term or phrase searching.

There were few policy-related articles that met our search and inclusion criteria. We feel strongly that evidence-based policy initiatives are important; but given the contextual issues, especially at the level of governmental decisions, interventions to improve the use of knowledge are not nearly as definable as in the smaller contexts of individual or organizational behaviour change.

Knowledge translation and fields related to implementation of interventions to promote the use of evidence in practice is challenged by the terminology used to name the field but also to describe interventions. This limits our ability to communicate and synthesize knowledge. This scoping review cast as wide a net as possible to include classification schemes. Due to the issue of language, we know this approach has missed things. Moving forward, this inventory should assist us in managing our terminology challenges.

## Conclusion

This scoping review provides an overview of schemes currently used to classify interventions. These can be used for evaluation and comparison and to guide validation of existing and emerging models. While the optimum approaches to using these classification schemes are not currently known nor which function best under which circumstances, they can provide a systematic approach with consistent terminology for characterizing interventions. Additional work is needed in applying these schemes optimally, with comparative evaluations, in order to realize benefits to intervention design and reporting.

## Additional files

Additional file 1:
**References that we were unable to find.**
Additional file 2: Table A1. Lists to classify interventions. **Table A2.** Taxonomies to classify interventions. **Table A3.** Frameworks to classify interventions. **Table A4.** Other classification systems for interventions.
