# Supplementary material for: A scoping review of classification schemes of interventions to promote and integrate evidence into practice in healthcare
Source: Implement Sci. 2015 Mar 3;10:27. doi: 10.1186/s13012-015-0220-6 (PMC4352247; doi:10.1186/s13012-015-0220-6)
Supplement: Additional file 1: — References that we were unable to find. [file 13012_2015_220_MOESM1_ESM.docx]

Additional file 1. References that we were unable to find.

Dunton GF, Cousineau M, Reynolds KD: **The intersection of public policy and health behavior theory in the physical activity arena.** J Phys Act Health 2010, **7** :S91-8.

Øvretveit J. Which Interventions are Effective for Improving Patient Safety? A Synthesis of Research and Policy Issues. Stockholm: WHO HEN, Copenhagen and MMC, Karolinska, 2010.

The Health Foundation. Quest for Quality and Improved Performance (QQUIP). http://www.health.org.uk/qquip.

White P: **PETeR: a universal model for health interventions**. 2010.
