# Supplementary material for: A scoping review of classification schemes of interventions to promote and integrate evidence into practice in healthcare
Source: Implement Sci. 2015 Mar 3;10:27. doi: 10.1186/s13012-015-0220-6 (PMC4352247; doi:10.1186/s13012-015-0220-6)
Supplement: Additional file 2: Table A1. — Lists to classify interventions. Table A2. Taxonomies to classify interventions. Table A3. Frameworks to classify interventions. Table A4. Other classification systems for interventions. [file 13012_2015_220_MOESM2_ESM.docx]

Additional File 2.

Table A1. Lists to classify interventions.

Table A2. Taxonomies to classify interventions.

Table A3. Frameworks to classify interventions.

Table A4. Other classification systems for interventions.

Table A1. Lists to classify interventions.

| **Classification scheme** | **Year** | **Objective as stated by the authors** |
| --- | --- | --- |
| Abraham 2011 [1] | 2010 | not reported |
| AHRQ [2] | 2013 | not reported |
| Albrecht 2013 [3] | 2013 | a checklist to operationalize the Workgroup for Intervention Development and Evaluation Research (WIDER) recommendations which provide a framework to identify and provide detailed reporting of the essential components of behavior change interventions in order to facilitate replication, further development, and scale-up of the interventions |
| Bartholomew 2011 [4] | 2011 | book designed to help plan health promotion programs using intervention mapping approach |
| CPHI 2001 [5] | 2001 | an environmental scan of research transfer strategies used by organizations involved with health or social research and policy that also have an emphasis on knowledge transfer |
| EPOC 2010 [6] | 2010 | data collection checklist for Cochrane reviews |
| Health Systems Evidence 2013 [7] | 2012 | a continuously updated repository of syntheses of research evidence about governance, financial and delivery arrangements within health systems, and about implementation strategies that can support change in health systems. |
| Powell 2012 [8] | 2012 | presents a consolidated compilation of discrete implementation strategies, based on a review of 205 sources published between 1995 and 2011. |

Table A2. Taxonomies to classify interventions.

| **Classification scheme** | **Year** | **Objective as stated by the authors** |
| --- | --- | --- |
| Abraham 2008 [9] | 2008 | to develop and extend existing lists of content components into a set of distinct, theory-linked definitions of behavior change techniques (BCTs) and, to test whether these definitions could be used to reliably identify techniques included in interventions on the basis of intervention descriptions |
| Carlson 2010 [10] | 2010 | to identify, categorize, and evaluate performance-based health outcomes reimbursement schemes between medical product manufacturers and payers |
| Dogherty 2010 [11] | 2010 | to examine the current state of knowledge surrounding the concept of facilitation as a role and process in the implementation of research findings within the nursing context |
| Embry 2008 [12] | 2008 | describes evidence-based kernels, fundamental units of behavioral influence that appear to underlie effective prevention and treatment for children, adults, and families |
| Evenboer 2012 [13] | 2012 | to present an empirically developed taxonomy of care for youth |
| Galbraith 2011 [14] | 2011 | process to identify core elements based on the creation of a taxonomy [of evidence-based behavioral interventions for HIV/AIDS prevention] developed from a review of the literature and a novel approach for describing core elements so they are simple, measureable, achievable, results-based and tested |
| Geller 1990[15] | 1990 | A framework for designing large-scale injury control programs and for evaluating the impact of such programs. |
| Gifford 2013 [16] | 2012 | to pilot a leadership intervention designed to influence nurses’ use of guideline recommendations when caring for patients with diabetic foot ulcers in home care nursing and proposing a taxonomy of leadership behaviors |
| Hardeman 2000 [17] | 2000 | to describe the interventions aimed at the prevention of weight gain; and to characterize the target behaviours; the psychological models underlying the interventions, behaviour change methods and modes of delivery; the methodological quality of the evaluation; the characteristics of the participants; and the outcomes of the studies |
| Lamb 2011 [18] | 2011 | to develop a classification system to characterize the major influential components of fall prevention interventions and promote consistency of reporting across international boundaries |
| Leeman 2007 [19] | 2007 | linked existing taxonomies with relevant theories to create a system for categorizing implementation methods |
| Lowe 2011 [20] | 2011 | of a classification system (or taxonomy) of interventions aiming to: (1) organize the range of interventions to improve prescribing for and medicines use by consumers in a meaningful way; (2) define the range of interventions available in order to improve sector-wide awareness and understanding; and (3) provide an organized entry point for decision makers to find evidence about the effectiveness of a wide range of interventions. |
| Mazza 2013 [21] | 2013 | was to draft an implementation taxonomy and to pilot its usefulness and feasibility as a tool for classifying implementation strategies |
| Michie 2012 [22] | 2012 | to develop a reliable taxonomy of behaviour change techniques (BCTs) used in interventions to reduce excessive alcohol consumption (not to treat alcohol dependence) |
| Michie 2008 [23] | 2008 | illustrate methods for developing an extensive list of behaviour change techniques (with definitions) and for linking techniques to theoretical constructs. |
| Michie 2011 [24] | 2011 | to extend the scope and improve the reliability of a 26-item taxonomy of behaviour change techniques developed by Abraham and Michie in order to optimize the reporting and scientific study of behaviour change interventions |
| Michie 2011 [25] | 2011 | to develop for the first time a reliable taxonomy of behaviour change techniques (BCTs) used within individual behavioural support for smoking cessation |
| Reisman 2005 [26] | 2005 | presents a taxonomy defining the field of transfer of technologies in its entirety and delineating all of its facets in a manner that is parsimonious yet discriminating |
| Schulz 2010 [27] | 2010 | present a taxonomy for describing intervention protocols designed to help researchers conceptualize needed elements of intervention protocols and to enhance both the internal (e.g., understanding the active ingredients of intervention components) and external validity (e.g., replicating studies in real world settings) of intervention research |
| Shojania 2004 [28] | 2004 | a critical analysis of the existing literature on quality improvement strategies for a selection of 20 disease and practice priorities |
| Taylor 2011 [29] | 2011 | develop a taxonomy of which contextual features which may be important determinants of the efficacy of patient safety practice interventions |
| Walter 2003 [30] | 2003 | a taxonomy of interventions to enhance the impact of research on public sector policy and practice which has been developed by the Research Unit for Research Utilization (RURU) at the University of St. Andrews |
| West 2006 [31] | 2006 | review on current and future tobacco control efforts |

Table A3. Frameworks to classify interventions.

| **Classification scheme** | **Year** | **Objective as stated by the authors** |
| --- | --- | --- |
| Best 2008 [32] | 2008 | to nurture common ground upon which to build a platform for translating what we know about cancer into what we do in practice and policy |
| Cane 2012 [33] | 2012 | to examine the content validity of the Theoretical Domains Framework to confirm optimal domain structure (number of domains), domain content (component constructs in each domain), and domain labels (most appropriate names that best reflected the content of the validated domain structure). |
| Century 2012 [34] | 2012 | a conceptual framework for describing aspects of implementation, a framework for describing the factors that affect implementation, and tools for measuring each |
| Cohen 2000 [35] | 2000 | proposes a new taxonomy of only two major categories of sexually transmitted disease/HIV preventive interventions |
| Czaja 2003 [36] | 2003 | an alternative strategy that facilitates decomposition of complex psychosocial and behavioral interventions into their basic observable elements |
| Damshroder 2009 [37] | 2009 | the Consolidated Framework For Implementation Research (CFIR) offers an overarching typology to promote implementation theory development and verification about what works where and why across multiple contexts. The CFIR will help advance implementation science by providing consistent taxonomy, terminology, and definitions on which a knowledge base of findings across multiple contexts can be built |
| Dixon 2010 [38] | 2010 | The Health Behaviour Change Competency Framework (HBCC) orders the competences described in the document Generic Health Behaviour Change: A Comprehensive Competency Framework into a hierarchy, to be used to develop training programs for health and other professionals |
| Dolan 2010 [39] | 2010 | outline 9 robust influences on human behaviour and underpinned by research from social psychology and behavioural economics |
| Dy 2011 [40] | 2011 | Develop and evaluate a framework for describing and classifying patient safety practices. |
| Goel 1996 [41] | 1996 | a conceptual framework in which to analyze factors that may affect retail pharmacy behaviors, and suggests strategies which might be used for changing pharmacy behaviors |
| Hendriks 2013 [42] | 2013 | to introduce a framework for the development and implementation of integrated public health policies |
| Lavis 2006 [43] | 2006 | develop a framework for assessing country-level efforts to link research to action. The main purpose of the framework is to inform country level dialogues about the domains to which attention could be directed in order to link research to action. |
| Michie 2011 [44] | 2011 | review existing frameworks of behavioural interventions and construct a framework of behaviour change interventions |
| Purdue 2005 [45] | 2005 | a conceptual overview of legal strategies, applicable at the federal, state, and local levels, that can be employed by healthcare providers, public health practitioners, legislators, and other policymakers for addressing the public health burden of cardiovascular disease |
| Stirman 2013 [46] | 2013 | to develop a coding scheme to characterize modifications made to evidence based interventions when they are implemented in contexts or with populations that differ from that in which they were originally developed or tested |

Table A4. Other classification systems for interventions.

| **Classification scheme** | **Year** | **Objective as stated by the authors** |
| --- | --- | --- |
| Greenhalgh 2004 [47] | 2004 | summary of the findings of a systematic literature review of the diffusion of service innovations, focused on health literature |
| Keller 2004 [48] | 2004 | introduces the revised Intervention Wheel and the evidence linking it to practice |
| Nuffeld 2007 [49] | 2007 | describes different kinds of intervention that may be used to promote public health by public authorities on an ‘intervention ladder’, from the least to the most coercive or intrusive measures |
| Proctor 2013 [50] | 2013 | propose specific standards for characterizing implementation strategies in sufficient detail |
| Ward 2010 [51] | 2010 | to produce a template to help researchers, practitioners and decision makers plan and evaluate initiatives for transferring knowledge into action |

Reference List

1. Abraham C, Kok G, Schaalma H, Luszczynska A: **Health Promotion.** In *The International Association of Applied Psychology Handbook of Applied Psychology.* Edited by Martin PR, Cheung FM, Knowles MC, Kyrios M, Overmier JB, Prieto JM. Oxford: Wiley-Blackwell.; 2011.

2. Agency for Healthcare Research and Quality-Patient Safety Network. Approach to Improving Safety. http://psnet.ahrq.gov/collection.aspx?taxonomyID=600 . 2013. Accessed 30 Jul 2013.

3. Albrecht L, Archibald M, Arseneau D, Scott SD: **Development of a checklist to assess the quality of reporting of knowledge translation interventions using the Workgroup for Intervention Development and Evaluation Research (WIDER) recommendations.** *Implement Sci* 2013, **8:** 52.

4. Bartholomew LK, Parcel GS, Kok G, Gottlieb N: *Planning Health Promotion Programs: Intervention Mapping*. San Francisco: Jossey-Bass; 2011.

5. Canadian Population Health Initiative. An environmental scan of research transfer strategies. 2001. Canadian Institute for Health Information.

6. Cochrane Effective Practice and Organisation of Care Group. EPOC resources for review authors. http://epoc.cochrane.org. 2010. Accessed 4 May 2012.

7. McMaster Health Forum. Health Systems Evidence. http://www.healthsystemsevidence.org/open-search.aspx . 2013. Accessed 3 Jan 2013.

8. Powell BJ, McMillen JC, Proctor EK, Carpenter CR, Griffey RT, Bunger AC, Glass JE, York JL: **A compilation of strategies for implementing clinical innovations in health and mental health.** *Med Care Res Rev* 2012, **69:** 123-157.

9. Abraham C, Michie S: **A taxonomy of behavior change techniques used in interventions.** *Health Psychol* 2008, **27:** 379-387.

10. Carlson JJ, Sullivan SD, Garrison LP, Neumann PJ, Veenstra DL: **Linking payment to health outcomes: a taxonomy and examination of performance-based reimbursement schemes between healthcare payers and manufacturers.** *Health Policy* 2010, **96:** 179-190.

11. Dogherty EJ, Harrison MB, Graham ID: **Facilitation as a role and process in achieving evidence-based practice in nursing: a focused review of concept and meaning.** *Worldviews Evid Based Nurs 2010,* **7**: 76-89.

12. Embry DD, Biglan A: **Evidence-based kernels: fundamental units of behavioral influence.** *Clin Child Fam Psychol Rev* 2008, **11:** 75-113.

13. Evenboer K, Huyghen AM, Tuinstra J, Knorth E, Reijneveld S: **A Taxonomy of Care for Youth: Results of an Empirical Development Procedure.** *Res Soc Work Pract* 2012, **22:** 637-646.

14. Galbraith JS, Herbst JH, Whittier DK, Jones PL, Smith BD, Uhl G, Fisher HH: **Taxonomy for strengthening the identification of core elements for evidence-based behavioral interventions for HIV/AIDS prevention.** *Health Educ Res* 2011, **26:** 872-885.

15. Geller ES, Berry TD, Ludwig TD, Evans RE, Gilmore MR, Clark SW: **A conceptual framework for developing and evaluating behavior change interventions for injury control.** *Health Educ Res* 1990, **5:** 125-137.

16. Gifford W, Davies B, Graham I, Tourangeau A, Woodend A, Lefebre N: **Developing Leadership Capacity for Guideline Use: A Pilot Cluster Randomized Control Trial.** *Worldviews Evid Based Nurs* 2013, **10:** 51-65.

17. Hardeman W, Griffin S, Johnston M, Kinmonth AL, Wareham NJ: **Interventions to prevent weight gain: a systematic review of psychological models and behaviour change methods.** *Int J Obes Relat Metab Disord* 2000, **24:** 131-143.

18. Lamb SE, Becker C, Gillespie LD, Smith JL, Finnegan S, Potter R, Pfeiffer K; ; Taxonomy Investigators: **Reporting of complex interventions in clinical trials: development of a taxonomy to classify and describe fall-prevention interventions.** *Trials* 2011, **12:** 125.

19. Leeman J, Baernholdt M, Sandelowski M: **Developing a theory-based taxonomy of methods for implementing change in practice.** *J Adv Nurs* 2007, **58:** 191-200.

20. Lowe D, Ryan R, Santesso N, Hill S: **Development of a taxonomy of interventions to organise the evidence on consumers' medicines use.** *Patient Educ Couns* 2011, **85:** e101-e107.

21. Mazza D, Bairstow P, Buchan H, Chakraborty S, VanHecke O, Grech C, Kunnamo I: **Refining a taxonomy for guideline implementation: results of an exercise in abstract classification.** *Implement Sci* 2013, **8:** 32.

22. Michie S, Whittington C, Hamoudi Z, Zarnani F, Tober G, West R: **Identification of behaviour change techniques to reduce excessive alcohol consumption.** *Addiction* 2012, **107:** 1431-1440.

23. Michie S, Johnston M, Francis JJ, Hardeman W, Eccles MP: **From Theory to Intervention: Mapping Theoretically Derived Behavioural Determinants to Behaviour Change Techniques.** *Appl Psychol* 2008, **57:** 660-680.

24. Michie S, Ashford S, Sniehotta FF, Dombrowski SU, Bishop A, French DP: **A refined taxonomy of behaviour change techniques to help people change their physical activity and healthy eating behaviours: the CALO-RE taxonomy.** *Psychol Health* 2011, **26:** 1479-1498.

25. Michie S, Hyder N, Walia A, West R: **Development of a taxonomy of behaviour change techniques used in individual behavioural support for smoking cessation.** *Addict Behav* 2011, **36:** 315-319.

26. Reisman A: **Transfer of technologies: a cross-disciplinary taxonomy.** *Omega 33* 2005, 189-202.

27. Schulz R, Czaja SJ, McKay JR, Ory MG, Belle SH: **Intervention taxonomy (ITAX): describing essential features of interventions.** *Am J Health Behav* 2010, **34:** 811-821.

28. Shojania KG, McDonald KM, Wachter RM, Owens DK. Closing The Quality Gap: A Critical Analysis of Quality Improvement Strategies, Volume 1—Series Overview and Methodology. Technical Review 9 (Contract No. 290-02-0017 to the Stanford University-UCSF Evidence-based Practices Center). 8-1-2004. Rockville, MD, Agency for Healthcare Research and Quality. AHRQ Publication No. 04-0051-1.

29. Taylor SL, Dy S, Foy R, Hempel S, McDonald KM, Ovretveit J, Pronovost PJ, Rubenstein LV, Wachter RM, Shekelle PG: **What context features might be important determinants of the effectiveness of patient safety practice interventions?** *BMJ Qual Saf* 2011, **20:** 611-617.

30. Walter I, Nutley S, Davies H. Developing a taxonomy of interventions used to increase the impact of research . 2003. University of St Andrews.

31. West R: **Tobacco control: present and future.** *Br Med Bull* 2006, **77-78:** 123-136.

32. Best A, Hiatt RA, Norman CD: **Knowledge integration: conceptualizing communications in cancer control systems.** *Patient Educ Couns* 2008, **71:** 319-327.

33. Cane J, O'Connor D, Michie S: **Validation of the theoretical domains framework for use in behaviour change and implementation research.** *Implement Sci* 2012, **7:** 37.

34. Century J, Cassata A, Rudnick M, Freeman C: **Measuring enactment of innovations and the factors that affect implementation and sustainability: moving toward common language and shared conceptual understanding.** *J Behav Health Serv Res* 2012, **39:** 343-361.

35. Cohen DA, Scribner R: **An STD/HIV prevention intervention framework.** *AIDS Patient Care STDS* 2000, **14:** 37-45.

36. Czaja SJ, Schulz R, Lee CC, Belle SH: **A methodology for describing and decomposing complex psychosocial and behavioral interventions.** *Psychol Aging* 2003, **18:** 385-395.

37. Damschroder LJ, Aron DC, Keith RE, Kirsh SR, Alexander JA, Lowery JC: **Fostering implementation of health services research findings into practice: a consolidated framework for advancing implementation science.** *Implement Sci* 2009, **4:** 50.

38. Dixon D, Johnston M. Health Behaviour Change Competency Framework: Competences to deliver interventions to change lifestyle behaviours that affect health. 1-46. 10-11-2010. NHS Health Scotland.

39. Dolan P, Hallsworth M, Halpern D, King D, Vlaev I. MINDSPACE; Influencing behaviour through public policy. 2010. Institute for Government, the Cabinet Office.

40. Dy SM, Taylor SL, Carr LH, Foy R, Pronovost PJ, Ovretveit J, Wachter RM, Rubenstein LV, Hempel S, McDonald KM, Shekelle PG: **A framework for classifying patient safety practices: results from an expert consensus process.** *BMJ Qual Saf* 2011, **20:** 618-624.

41. Goel P, Ross-Degnan D, Berman P, Soumerai S: **Retail pharmacies in developing countries: a behavior and intervention framework.** *Soc Sci Med* 1996, **42:** 1155-1161.

42. Hendriks AM, Jansen MW, Gubbels JS, De Vries NK, Paulussen T, Kremers SP: **Proposing a conceptual framework for integrated local public health policy, applied to childhood obesity - the behavior change ball.** *Implement Sci* 2013, **8:** 46.

43. Lavis JN, Lomas J, Hamid M, Sewankambo NK: **Assessing country-level efforts to link research to action.** *Bull World Health Organ* 2006, **84:** 620-628.

44. Michie S, van Stralen MM, West R: **The behaviour change wheel: A new method for characterising and designing behaviour change interventions.** *Implement Sci* 2011, **6:** 42.

45. Perdue WC, Mensah GA, Goodman RA, Moulton AD: **A legal framework for preventing cardiovascular diseases.** *Am J Prev Med* 2005, **29:** 139-145.

46. Stirman SW, Miller CJ, Toder K, Calloway A: **Development of a framework and coding system for modifications and adaptations of evidence-based interventions.** *Implement Sci* 2013, **8:** 65.

47. Greenhalgh T, Robert G, Macfarlane F, Bate P, Kyriakidou O: **Diffusion of innovations in service organizations: systematic review and recommendations.** *Milbank Q* 2004, **82:** 581-629.

48. Keller LO, Strohschein S, Lia-Hoagberg B, Schaffer MA: **Population-based public health interventions: practice-based and evidence-supported. Part I.** *Public Health Nurs* 2004, **21:** 453-468.

49. Nuffield Council on Bioethics. Public health: ethical issues:a guide to the report. 2007. Nuffield Council on Bioethics.

50. Proctor EK, Powell BJ, McMillen CJ: **Implementation strategies: recommendations for specifying and reporting.** *Implement Sci* 2013, **8:** 139.

51. Ward V, Smith S, Carruthers S, Hamer S, House A. Knowledge brokering: Exploring the process of transferring knowledge into action. 2010. UK, University of Leeds.
